# Non-traditional Environmental and Cultural Determinants of Cardiovascular Disease in India: A Narrative Review

**DOI:** 10.7759/cureus.105876

**Published:** 2026-03-26

**Authors:** Ashok Soota, Yogesh Shouche, Lavanya Garady, Srikanth KV, Komal Prasad Chandrachari, Sanketh V Sharma, Subrahmanya Kumar Kukkupuni, Azad Devyani, Amritha S, Ahalya P Gopi

**Affiliations:** 1 Information Technology, SKAN Research Trust, Bengaluru, IND; 2 Microbiology, SKAN Research Trust, Bengaluru, IND; 3 Public Health Sciences, SKAN Research Trust, Bengaluru, IND; 4 Cardiology, Narayana Institute of Cardiac Sciences, Bengaluru, IND; 5 Neurosurgery, Mazumdar Shaw Medical Centre, Narayana Health City, Bengaluru, IND; 6 Ayurvedic Medicine, The University of Trans-Disciplinary Health Sciences and Technology, Bengaluru, IND; 7 Centre for Ayurveda Biology and Holistic Nutrition, The University of Trans-Disciplinary Health Sciences and Technology, Bengaluru, IND

**Keywords:** cardiovascular diseases, cultural risk factors for cvds, emerging risk factors, environmental factors for cvds, heart attack, stroke

## Abstract

This literature review identifies non-traditional environmental and cultural risk factors for cardiovascular diseases (CVDs) and explores their role in predictability. Available references are primarily concentrated on traditional risk factors for CVD, leaving a gap in understanding the role of new environmental and cultural variables that contribute to the disease, particularly in low- and middle-income countries (LMICs), like India. Identifying these risk factors can help focus on primordial prevention, leading to better health outcomes, lower healthcare expenditures, and improved quality of life. Given that CVD continues to be the world's leading cause of mortality and has a disproportionate burden in LMICs, the study holds significance within the larger field of epidemiology. The comparative focus is on India, where CVD mortality rates are notably high, and the interplay of cultural practices and environmental exposures is critical.

An in-depth review was conducted using selected English articles published in databases such as PubMed, Scopus, and Science Direct over a period of 15 years, from 2010 to 2024. Terms associated with environmental and cultural risk factors for CVD were added to the search strategy. MeSH (Medical Subject Headings) terms, such as myocardial infarction (MI), heat shock, and heart disease risk factors, as well as indoor air pollution, and Boolean operators, were also used to improve the search outcome. Review articles and cross-referenced articles were also considered in the review to compile all possible risk factors. Major factors discussed under environmental factors were air pollution, noise pollution, artificial light exposure, and seasonal changes, whereas the cultural factors taken into consideration were food habits, cooking oil used, habitual tobacco use, western migration, and ethnicity.

The review was conducted over a period of four months, from March to June 2024, and included both global and Indian articles, as evidence from the Indian context alone was limited. After removing duplicates and excluding articles without full text or relevance to the objectives, 97 articles were included in the final review. As suggested by the evidence from the references, there is a dose-response link between environmental variables, such as noise and air pollution, and cardiovascular disorders. The level of exposure is also increased by the use of biomass fuel and indoor cooking. Exposure to artificial light primarily alters the body's circadian rhythm, leading to imbalances in CVD-related risk factors over time. Although the impact of seasonal variations is unclear, it must be investigated.

According to Indian culture, our dietary practices have a detrimental effect on direct risk factors for CVD, such as diabetes and hyperlipidemia. The type and high quantity of cooking oil used, as well as reheating and deep-frying cooking patterns, have also been linked to an increased risk of atherosclerosis and adverse cardiovascular outcomes. There is a critical gap in evidence generation regarding non-traditional risk factors in India, which emphasizes the need for more longitudinal studies and risk prediction models that incorporate environmental and cultural determinants of cardiovascular risk assessment in the Indian population.

## Introduction and background

Background

Cardiovascular disease (CVD) is a common term used to describe a set of conditions that affect the routine functioning of the heart and blood vessels, mainly including coronary heart disease, stroke, and TIAs (transient ischemic attacks, also called mini-strokes). CVDs are mainly caused by the formation of fatty buildups, called plaques, inside the blood vessels, which increase the risk of blood clots [1,2]. As per the WHO fact sheet data, CVDs have become the leading cause of death globally, contributing around 32% of total deaths. Of these, 85% of deaths are attributed to heart attacks and stroke [3]. Over the past 20 years, there has been more than a 20% increase in disability-adjusted life years (DALYs) associated with CVDs, as per the World Heart Federation data [4].

Around 80% of total CVD deaths occur in low- and middle-income countries (LMICs), and the risk factor burden is increasing due to the ongoing epidemiological transition [5,6]. Considering the Indian scenario, the burden is higher when compared to global estimates, such as the age-standardized death rate, which is around 282 deaths per 100,000, compared with global levels (233 deaths per 100,000) [7,8]. The major known risk factors for CVDs are grouped and categorized in many ways, and one of the broad classifications is into modifiable and non-modifiable risk factors. Non-modifiable risk factors include age, gender, race, and family history, whereas modifiable risk factors encompass dietary behaviors, physical activity, habits, and lifestyle-related factors [9,10]. Among the modifiable risk factors, diabetes, elevated cholesterol levels, and low physical activity are reported as major individual contributors to CVD in India [11,12]. Beyond these conventional factors, several newly identified and emerging determinants may directly or indirectly contribute to the development of CVDs. Environmental and culture-related factors are now considered among the emerging causes that can affect cardiovascular health, owing to demographic transition, urbanization, and climate change [13-15].

Identifying and gathering more evidence on emerging risk factors can help predict this multifactorial condition - especially myocardial infarction (MI) and stroke - in a more reliable manner, thereby facilitating the prevention of these risk factors and reducing their effect size, leading to improved quality of life, reduced catastrophic health expenditure, and mitigated caregiver burnout.

Objective

The main objective of this study is to identify various environmental and cultural risk factors, their association with CVDs, and their role in prediction.

## Review

Methodology

A systematic funneling strategy was adopted to identify relevant articles for this narrative review on non-traditional environmental and cultural risk factors associated with CVDs. Electronic databases like PubMed, followed by Scopus, SpringerLink, and ScienceDirect, were primarily used to identify relevant references. Articles from Google Scholar and relevant grey literature were also included to ensure comprehensive coverage of relevant studies.

The article search was limited to English-language literature, including cross-sectional studies, case-control studies, observational studies, cohort studies, and relevant review articles published between 2010 and 2024. The literature review was conducted over a period of four months, from March to June 2024, and included both global and Indian articles, as evidence from the Indian context alone was limited.

The initial search started with risk factors for CVDs and then narrowed down to keywords such as "emerging risk factors", "cardiovascular diseases", "heart attack", "stroke", "environmental factors for CVDs", and "cultural risk factors for CVDs". General and advanced searches using Boolean operators were conducted in PubMed, and the MeSH database was used to identify keywords ("myocardial infarction", "heat shock", "heart disease risk factors", "indoor air pollution", etc.). Cross-referencing was also conducted using the reference lists of relevant articles to identify additional studies that may not have been captured in the initial search.

Studies examining environmental or cultural determinants of CVDs and providing evidence on their potential association with cardiovascular outcomes were included in the review. Articles were excluded if they were not available in full text, were not published in English, or were not relevant to the study objectives. Initially, we identified and enlisted all possible risk factors associated with CVDs under two categories: environmental and cultural. Following this, an in-depth review was conducted for individual risk factors to explore their potential associations with CVDs, especially heart attack and stroke.

**Figure 1:**
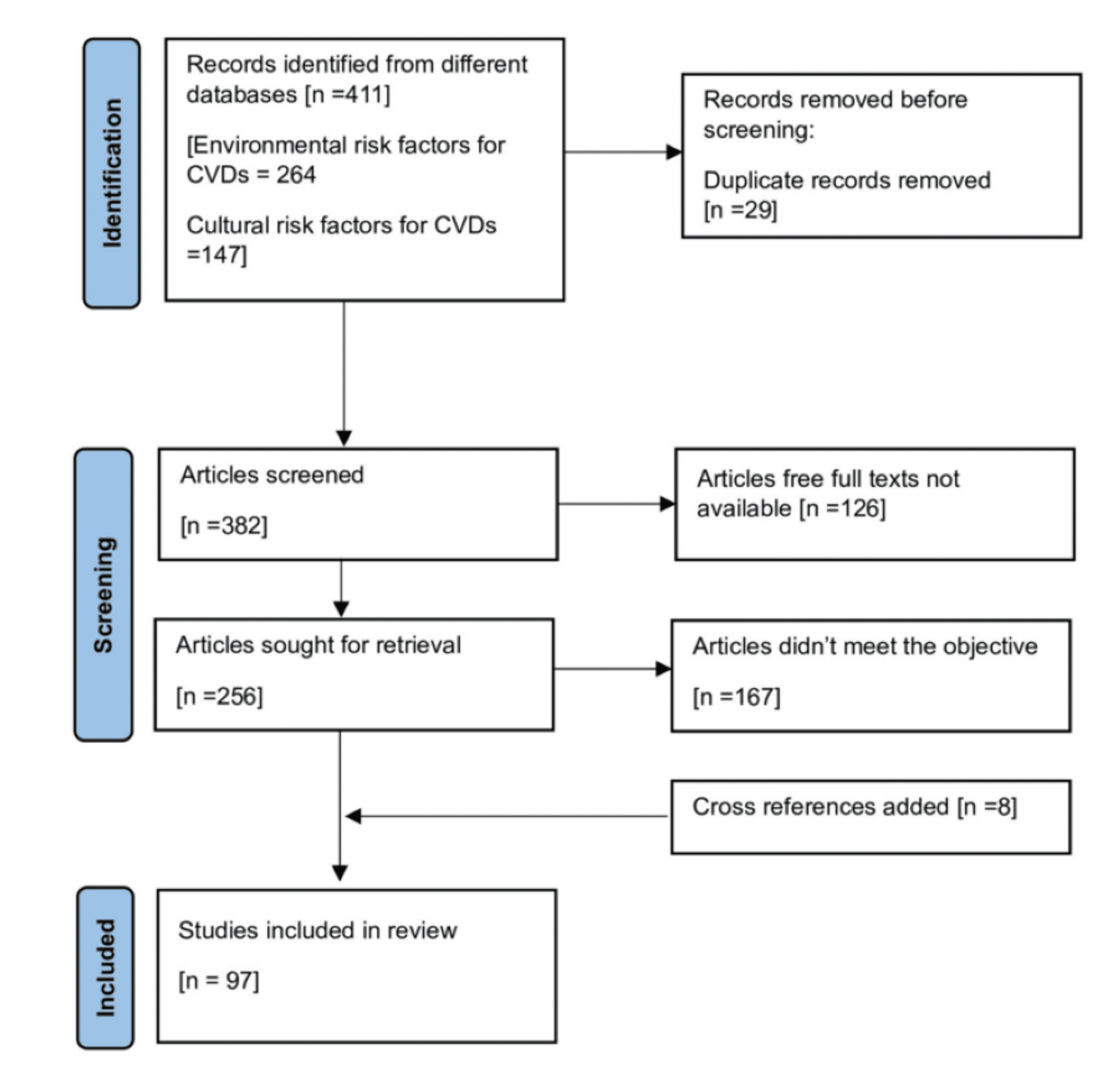
PRISMA schematic representation of search strategy followed for finding the articles as per the objective. PRISMA 2020 open access reporting guideline format [16]. PRISMA: Preferred Reporting Items for Systematic Reviews and Meta-Analyses

Although this is not a systematic review, we have referred to the open-access PRISMA 2020 reporting guidelines to structure the search strategy and guide the selection of articles for inclusion [16]. After removing duplicates and excluding studies that did not meet the inclusion criteria, 97 articles were included in the final review. The article selection process is illustrated in Figure 1 using a PRISMA flow diagram. Formal systematic review processes, such as dual screening, detailed search strings, quality assessment, and risk of bias evaluation, were not performed, as this is a narrative review.

Results

The literature review mainly concentrated on the emerging role of environmental and cultural factors in CVD incidence among the Indian population. Major factors identified and discussed under environmental factors were air pollution, noise pollution, artificial light exposure, and seasonal changes, whereas the cultural factors taken into consideration were food habits, cooking oil used, habitual tobacco use, western migration, and ethnicity.

Environmental factors, such as air pollution and noise pollution, were found to have a dose-response relationship with CVDs, which has been demonstrated in previously published studies. The practice of indoor cooking and the use of biomass fuel also increase the level of exposure. Artificial light exposure mainly affects the diurnal rhythm of the body, and, in the long run, this indirect effect causes an imbalance in CVD-related risk factors. The effect of seasonal changes is not very clear but needs to be explored.

As per Indian culture, the food habits we follow themselves have a negative impact on direct CVD risk factors, such as hyperlipidemia and diabetes. The cooking oil mostly consumed in India is palm oil, as it is comparatively cheaper and easily available, and it has a relatively higher saturated fatty acid (SFA) content, which is assumed to increase the risk of atherosclerosis and overall cardiovascular outcomes.

Discussion

Environmental Factors

In day-to-day life, people are continuously exposed to variations in environmental factors, such as geographical, seasonal, or occupation-related factors. Some of the identified environmental risk factors for CVDs include air pollution, noise pollution, light exposure, proximity to traffic, seasonal changes (such as heat stress and sunlight exposure), geographical differences (such as altitude changes and green spaces), and occupational risk factors (such as fuel smoke exposure from biomass and kerosene) [17]. According to Global Burden of Diseases (GBD) data 2019, air pollution is the fourth leading cause of death [17-19]. Indian studies indicate that tobacco smoke exposure in public spaces impacts CVD risk, despite all these factors [20].

Air pollution: WHO defines air pollution as “the contamination of the indoor or outdoor environment by any chemical, physical, or biological agent that modifies the natural characteristics of the atmosphere.” Along with the enhancement of technologies in the growing world, outdoor and indoor air pollution has evolved to become one of the major causes of global mortality and morbidity. More than 99% of the global population has been breathing air containing higher levels of pollutants, exceeding WHO guideline limits [18]. Household air pollution also contributes to around 3.2 million deaths globally every year, with approximately 32% of these deaths due to ischemic heart disease (IHD) and 23% due to stroke [21].

Air containing particulate matter with a size <2.5 µm, NO_2_, CO, SO_2_, and O_3_ is associated with CVD, especially stroke. Various cohort studies conducted in Denmark, the USA, and Japan have shown a positive association between ambient air pollution and increased risk of IHD and stroke [22-28]. The association between daily concentrations of pollutants, such as NO2, and the incidence of acute myocardial infarction (AMI) has been established even among non-smokers [29]. An Indian study by Sajith Kumar et al. also shows that the state-wise air pollution-attributed cardiovascular disease (APACVD) burden increases with indirect economic development indices, including annual new motor vehicle registrations and the number of functional factories [30]. Along with this, increased biomass fuel consumption in Indian households for cooking is also positively associated with cardiovascular risk factors, such as carotid intima-media thickness (CIMT), according to a South Indian cohort study [31].

Another factor that can be considered under air pollutants is secondhand tobacco smoke (SHS) exposure. According to WHO and CDC data, SHS exposure can increase the risk of heart attack and stroke by around 30% among non-smokers [32-34]. A prospective study conducted among non-smoking women in Japan reported an increased incidence of IHD and stroke associated with the husband’s smoking status [35]. Findings from the Global Adult Tobacco Survey (GATS) Round 2 in India revealed that SHS exposure is prevalent in approximately 40% of Indian households and 23% of public spaces (including government offices, healthcare facilities, public transportation, and restaurants), with workplace exposure in particular being around 30% [36]. The cardiovascular effect of this is assumed to be huge among the Indian population, even though studies examining it remain limited within this demographic.

**Figure 2:**
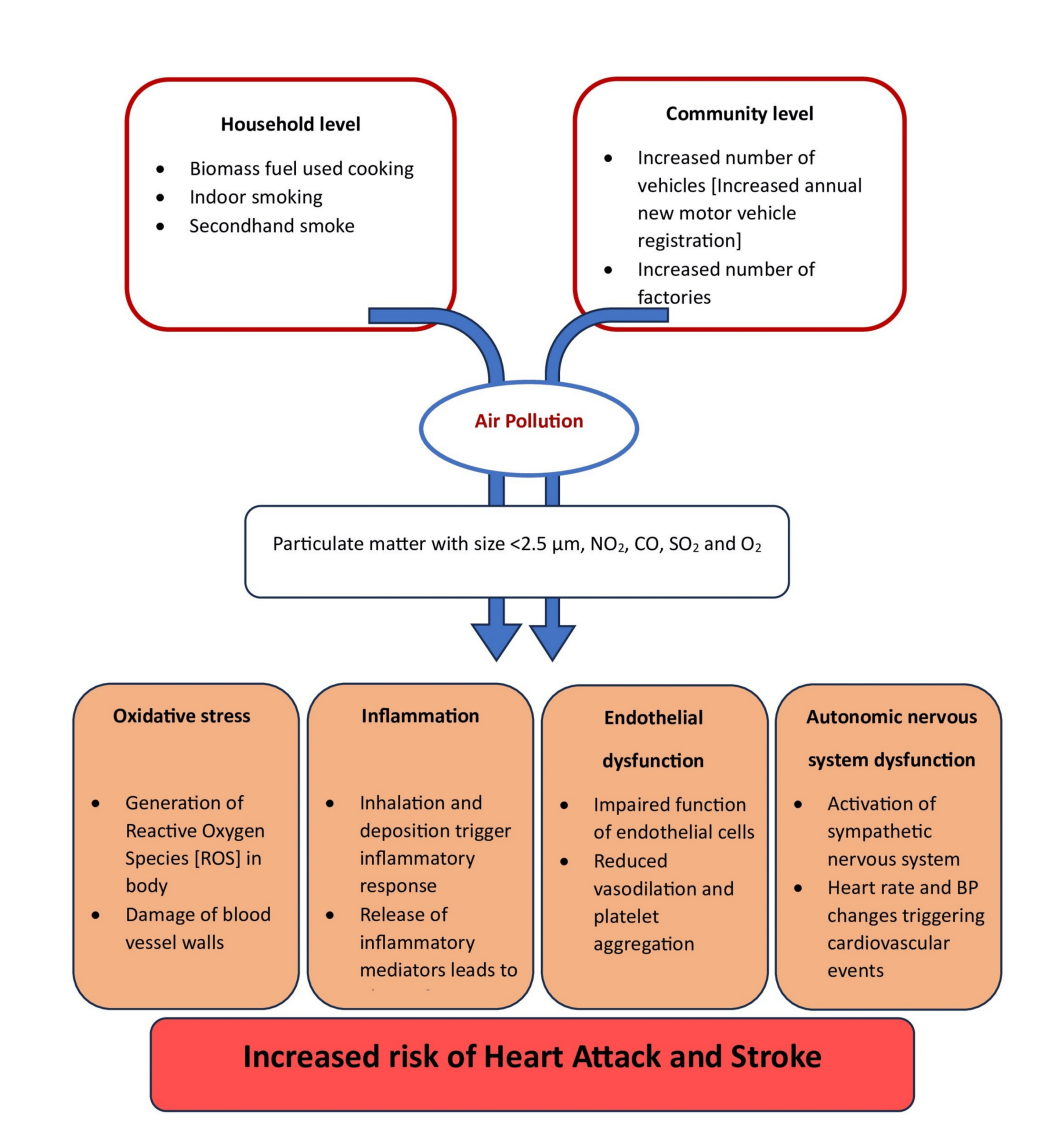
Household and community level air pollution exposure and its overall effect. The image was created by the authors using Microsoft Word (Microsoft Corporation, Redmond, WA, USA).

The practice of cooking using biomass fuel is common in various Indian states, especially in the Northeastern states. According to the Centre for Science and Environment data published in December 2023, around 40% of the Indian population continues to use biomass fuels, such as firewood, animal dung, crop residues, and kerosene, for cooking, despite continuous efforts by the Government to improve access to clean fuels [37]. A study conducted by IIT Mandi in the Northeastern states corroborates this, providing an estimate of the potential number of years lost in the population due to premature mortality from ill health resulting from indoor air pollution [38]. According to the WHO household air pollution exposure data, 12% of IHD and stroke deaths can be attributed to solid fuel and kerosene consumption at home [21]. Exposure to biomass fuel increased cardiovascular risk by around four times compared to those who used liquefied petroleum gas (LPG), according to a cross-sectional study conducted in Thailand. The association was significant after adjusting for gender, age, cigarette smoke, SHS, and exposure to other sources of air pollution [39]. In an Indian study conducted in 2018 in Karnataka, cardiovascular risk was six times higher among those exposed to biomass fuel while cooking, compared to those who were not [40]. The effect on health can also change depending on the duration of exposure and is often expected to have a dose-response relationship, which requires further exploration and evidence generation. Quantifying the level of exposure to the pollutants described above can be one of the factors adding value to cardiovascular risk prediction tools (Figure 2).

Noise pollution:* *Noise is an unavoidable exposure nowadays, which can cause multiple short-term and long-term health effects, such as auditory impairments, sleep disturbances, adverse cardiovascular outcomes, and a decline in overall well-being. The primary sources of environmental noise exposure are road, railway, and air traffic, construction sites, and other factories; however, there is limited evidence on the global health impacts of environmental noise pollution [41].

Harvard Health led a five-year follow-up study to assess the association between home-based exposure to traffic and aviation noise and cardiovascular effects. After controlling for other known risk factors, it was found that, for every 5 dB increase in the average 24-hour noise level, there is a 30% increase in the likelihood of stroke, heart attack, and other major health conditions [42]. Similarly, a follow-up study conducted in Germany from 2012 to 2022 (Gutenberg Health Study) identified noise irritation as an independent risk factor for various CVD events, which are source-specific and time-specific. Considering the distinct sources of noise, neighborhood noise has a 15% higher risk compared to noise from road traffic, aircraft, railway, and industrial sources, with its impact being 1.3 times greater during sleep periods than during the daytime [43]. Another study conducted in New Jersey found that 5% of heart attack hospitalizations were attributable to higher noise exposure in residential areas, with an average of 65 dB or higher throughout the day [44]. Additionally, different types of transportation noise exposure have been associated with all-cause or cause-specific CVD mortality [45]. A cross-sectional study in North India also reported that cardiovascular risk increases by 2.25 times with each 5 dB increase in traffic noise levels [46].

Based on the data described above, exposure to noise above the prescribed threshold has been shown to have various cardiovascular effects. However, the impact depends on several factors, such as the source of exposure, incremental rise, day- or night-time exposures, duration of exposure, and individual characteristics, including age, gender, and other physical and psychosocial environments. Assessment and compilation of all these individual factors can contribute to the predictive role and significance/weightage of noise exposure in CVD risk assessments.

Artificial light exposure: Light exposure might be day or night, natural or artificial, with varying impacts on the human body. Although sunlight exposure provides a protective function, the effect varies according to duration and intensity. A cohort study conducted in the UK assessed the relationship between daylight exposure and the incidence of heart failure. Healthy participants aged 40-70 years were enrolled during 2006-2010, with an average follow-up time of 12 years. The results showed a nonlinear, J-shaped association between self-reported outdoor light exposure time and heart failure outcomes. Participants who were exposed for an average of 1-2.5 hours per day had a lower risk of heart failure compared to the other two groups, indicating that both low and high exposure to outdoor light is associated with higher risks [47]. Similarly, a sub-cohort study from the UK Biobank also reported an increased risk of MI and stroke among those who had an average daylight exposure of 3.5 hours or more [48].

The invention of electric lights brought a revolutionary change across the globe, making human life safer and more convenient, especially at night. It facilitated the continuation of human activities without interruption, increasing productivity across different sectors [49]. According to data published by the International Dark-Sky Association in 2016, over 80% of the global population is exposed to light-polluted skies because of industrial developments worldwide. Such excessive exposure can affect the natural diurnal rhythm, as all living species on Earth require an optimal amount of darkness for survival. In humans, studies show that light exposure at night can suppress melatonin secretion and increase heart rate, which prevents the body from getting enough rest, and can also interfere with insulin metabolism, leading to complications such as sleep and depressive disorders, obesity, diabetes, cancer, and even CVDs [50-53]. The Chicago Healthy Ageing Study, conducted among individuals over 60 years of age, also supports this and reported hypertension among exposed participants [54]. A cross-sectional study conducted in South India shows that increased nighttime light intensity (NTLI) due to urbanization is positively associated with a higher risk of CVDs compared to those with less exposure [55].

Increased artificial light exposure during the night increases stroke risk by affecting blood flow to the brain. According to a cohort study conducted in China among 28,302 participants, the adjusted hazard ratio was 1.43 in the light-exposed group, showing a 43% elevated risk of stroke compared to the baseline group [56]. Further research is required to generate evidence on the direct effect of artificial light on various CVDs. Existing evidence suggests that artificial light exposure, particularly when it disrupts the circadian rhythm, may not directly affect CVDs but can influence multiple CVD risk factors, as revealed by various studies conducted across different regions.

Seasonal changes: Seasonal changes, which include winter and summer peaks, heat stress, humidity, etc., can induce bodily alterations, including variations in biomarker levels. Similarly, seasonal peaks associated with the incidence of CVDs have also been observed. 

The Tromsø study (1979-2008), a population-based cohort study conducted in the Arctic region during the winter peak, at around -3 °C, found that most cardiovascular risk factors, such as systolic and diastolic blood pressure, heart rate, body weight, total cholesterol (TC), and high-density lipoprotein cholesterol (HDL-C), showed a strongly associated seasonal pattern, peaking during winter. In contrast, triglycerides (TG) peaked in autumn, while C-reactive protein and fibrinogen showed their highest levels in spring. However, this study was conducted within the Arctic Circle, where the reported extreme temperature during the winter peak was around -3 °C [57]. 

Similarly, a cohort study conducted in China (2012-2018) among adults over 65 years showed an inverse relationship between average annual temperature and humidity and CVD risk. For every 1 °C rise in average annual temperature, there was a reported reduction in hypertension (3%), heart disease (6%), and stroke (5%), with an average annual temperature of 16 °C and humidity of 71% [58]. Despite this, extreme heat conditions can also cause various cardiovascular effects when combined with high relative humidity, as hot temperatures can affect blood pressure and thereby result in CVD-related hospitalizations [59]. A US-based open cohort study, conducted from 2000-2016, reported that long-term exposure to summer-specific humidity was associated with higher CVD hospitalization rates. The average summer-specific humidity reported was 12.0 g of water vapor per kg of dry air, and the temperature was around 30 °C [60].

The GBD study on cardiovascular risks also reported that non-optimal temperature (both hot and cold), even in milder variations, can contribute to increased cardiovascular mortality. This variation also depends on geographic areas and the ideal climatic conditions pertaining to each specific region. For example, heat-related mortalities are more common in Eastern Europe, whereas cold-related mortalities are more frequent in Sub-Saharan Africa [5,61,62]. This seasonal effect is more evident in populations living in moderate climatic conditions, with additional factors, such as air pollution and seasonal peak-induced infections, affecting its intensity [63].

As extreme seasonal temperature fluctuations are less pronounced in India compared to the regions mentioned above, the available literature examining the seasonal influence on CVD burden in the country remains limited. A retrospective cross-sectional study was conducted across all HMIS-registered public, private, rural, and urban health facilities of 36 states and union territories (UTs) of India in 2022 and reported a seasonal variation in the number of adolescent/adult deaths due to heart disease or hypertension. The mean seasonal mortality was highest in the winter season (January and February; mean temperature 20.91 °C), followed by post-monsoon (October to December; mean temperature 23.59 °C), with the lowest mean mortality during pre-monsoon (March to May; mean temperature 27.91 °C) [64].

Cultural-Related Factors

Food habits:* *Dietary patterns have a significant impact on carbohydrate, lipid, and glucose metabolism, which are some of the conventional risk factors for CVDs. In India, despite nearly half the population following a vegetarian diet, CVD prevalence is comparable to or even higher than that in Western countries. The timing of food intake, along with dietary patterns that include consumption of components such as excess carbohydrates, dairy products (cheese, butter, ghee), and oils, accompanied by a non-vegetarian diet, can contribute to the development of metabolic imbalances [65]. Meal timing and frequency, as well as intermittent fasting, are described below.

Along with composition, meal timing and frequency are also very important. Data from the NutriNet-Santé cohort study conducted in France, including more than 100,000 participants, showed that early breakfast (before 9 a.m.) and early dinner (before 9 p.m.), along with long night fasting, reduce CVD risk [66,67]. Studies worldwide have shown mixed results regarding the number of meals per day. The Seasonal Variation of Blood Cholesterol Study (1994-1998), conducted in the US, reported that more eating episodes in a day (four or more) are associated with a lower risk of CVDs compared to fewer episodes, after adjusting for age, sex, total energy intake, and physical activity - findings comparable with those of the National Health and Nutrition Examination Survey (NHANES) conducted between 1999 and 2014 [68,69]. Several cross-sectional studies support similar findings [70,71], whereas a cohort study conducted in the US reported that more than three standard eating occasions per day are associated with a higher risk of gaining 5 kg over a 10-year follow-up period [72]. Another prospective study among 24,011 US adults over 40 years of age shows that skipping breakfast or eating only one meal per day is associated with a higher risk of CVD mortality [73]. In the Indian context, the majority of the population traditionally follows the pattern of three meals per day: breakfast, lunch, and dinner. However, according to a report published jointly by the Food and Agriculture Organization of the United Nations (FAO), International Fund for Agricultural Development (IFAD), United Nations Children’s Fund (UNICEF), World Food Programme (WFP), and World Health Organization (WHO), over 50% of the Indian population cannot afford a healthy diet, indicating that the average meal frequency of most Indians is less than three meals per day - potentially contributing to higher CVD prevalence in India [74]. Further research is needed to establish a clearer relationship between meal frequency and CVD risk, as evidence in the Indian context remains limited.

Recently, intermittent fasting, preferably limited to a time window of 8-12 hours, has been popularized due to the assumption that this habit can help control lipid levels as well as weight gain [75]. According to reports published by the American Heart Association (AHA), based on a study among 20,000 US adults, CVD risk is higher among those who follow a time-restricted eating pattern of less than eight hours per day compared to those who follow a 12-16-hour pattern [76]. While evidence on the actual effect of intermittent fasting on CVDs is limited, available studies suggest a J-shaped association. Time-restricted eating within a 12-16-hour window may be protective against CVD compared to both shorter (eight hours or less) and longer (more than 16 hours) fasting periods.

Dietary composition: The Indian diet is based predominantly on starchy foods, including various sweets, with carbohydrates constituting more than 60% of total dietary intake. This can cause hyperglycemia and hyperlipidemia, along with low HDL levels. According to current evidence, diabetes and hypercholesterolemia are conventional risk factors for CVDs, which are caused by higher carbohydrate and fat intake [77]. This association is supported by a higher incidence of non-alcoholic fatty liver disease (NAFLD) among the Indian population, which is also linked to a high-carbohydrate diet exacerbated by a slightly higher genetic predisposition to insulin resistance among Asian Indian ethnic groups [78].

Similar to carbohydrate and protein components, dietary intake of fats also depends on traditional and cultural practices to which a particular community is attached. The Fat Study Reports (2015-16), published by the National Institute of Nutrition, revealed that the average daily intake of visible fat among the population of seven major cities in India was around 33 g/day, which is higher than the Indian Council of Medical Research (ICMR) recommended level of 20 g/day [79]. Later, the What India Eats report published in 2021 updated that overall fat intake among the urban population in India is around 52 g/day, which is higher than rural intake (36 g/day) [80].

A cross-sectional study comparing the prevalence of vegetarian and non-vegetarian diets between South Asia and the US reported that, compared to 3% of the US population, one-third of the South Asian population followed a vegetarian diet. However, US vegetarian diets were more consistent and healthier, and vegetarian groups in both populations had lower cardiovascular risks compared to non-vegetarian groups. The Indian diet pattern is also rich in carbohydrates and fats but poor in protein content, which further increases CVD risk, even though meat consumption is comparatively low. Evidence also suggests that cooking styles such as deep frying and full boiling result in the loss of major micronutrients [7,81].

Cooking oil used: In the diet, the type and quantity of oil used for cooking play an important role, as they can affect cholesterol levels and related lipid metabolism. The type of cooking oil used and the quantity of oil used are described below.

Considering better heart health, cooking oils containing more unsaturated fatty acids (UFAs) (monounsaturated and polyunsaturated) can better maintain the lipid profile compared to SFAs. Based on a systematic review and meta-analysis including 13 varieties of cooking oils and fats (safflower, sunflower, rapeseed, hempseed, flaxseed, corn, olive, soybean, palm, coconut oil, and beef fat, lard, and butter), it was found that safflower oil is the most effective in reducing TC and low-density lipoprotein cholesterol (LDL-C), followed by rapeseed and sunflower oil. Soybean oil is more effective in reducing TG, while coconut oil can better improve HDL-C. All vegetable oils were more cardioprotective compared to animal fats, oils, and butter. Canola, which belongs to the rapeseed family, also has improved glycemic control properties [82,83]. The Indian and global culinary contexts differ, since many Indian dishes require extensive stir-frying and oil-heating techniques. This necessitates the use of cooking oils that not only possess a high content of UFAs but are also stable and do not release toxic byproducts during prolonged heating. Similar to rapeseed oil, mustard oil, which is commonly used in India, has a favorable linoleic acid (LA) to alpha-linolenic acid (ALA) ratio (LA/ALA ratio), low SFAs, and high monounsaturated fatty acid (MUFA) content, along with relative stability [84]. Coconut oil is also considered to have a less detrimental effect on the lipid profile compared to ghee, palm oils, and other refined oils, but not as much as mustard oil [85]. Peanut oil is widely used in Indian cooking, and studies indicate that its relatively high smoke point makes it appropriate for high-temperature cooking, with potential beneficial effects on HDL-C [86].

Excess oil consumption has been proven to have a higher CVD risk, and Indian cuisine contains many varieties of fried foods that use more oil and ghee. As per current statistics, being comparatively cheap, palm oil is the most consumed oil among the Indian population, despite its negative effects. The domestic consumption of palm oil crossed 8 million metric tons in 2023, which is approximately 38% of the total edible oil consumption, as revealed by the Asian Palm Oil Alliance (APOA) [87]. Practices like frying and the reuse of heated oils in households, as well as in commercial establishments, also cause an increased risk for CVDs. According to a case-control study conducted on premature heart disease in India, the case group reported a significantly higher intake of fried food items, both shallow- and deep-fried [88]. As outlined in the ICMR guidelines on oil usage, repeated heating and storage of used cooking oils can oxidize the polyunsaturated fatty acids (PUFAs) within the oil and lead to the release of various harmful compounds [89,90].

Habitual tobacco use: The causal association between smoking and CVD risk has been established by scientific communities. In response, various government and non-government bodies have implemented initiatives aimed at spreading awareness and, consequently, cutting down on this unhealthy habit. Even though smoking prevalence has reduced in the last few years, the use of smokeless tobacco (SLT) is still prevalent [91]. As per GATS 2 results, SLT usage (21%) is double the prevalence of smoking (10%), especially among tribal populations, as these habits have become a part of their culture, traditions, and geographic specialties [92,93]. This higher usage is also associated with lower educational and socio-economic status [94].

Western migration: Human migration usually happens either internally within a country, from rural to urban regions, or internationally, often towards more economically developed nations. The objective is mostly for better education, job opportunities, and a more financially stable life. It may start with a single individual, followed by the relocation of the entire family. Migration involves changes in the habitat of a person, including physical and social environment, food habits, and cultures. Introduction to this new habitat, to which the individual is not adapted, can result in many lifestyle changes and dietary patterns. As the genetic makeup and inborn metabolism are not compatible with the Western lifestyle and food habits, the immigrant population reports a higher prevalence of CVDs and associated risk factors. A literature review conducted on the CVD risk of South Asian immigrants (SAIs) supported these facts [95]. Even though the obesity rate is comparatively low, diabetes, CVDs, and related mortality are more common among Asian-American migrant populations [96].

Ethnicity: Recent studies show that ethnicity also has some effect on CVD risk. The genetic makeup of a person is attributed to the ethnic group they belong to, which can further impact their body metabolism. Blacks and South Asians are found to have a higher CVD risk when compared to White and Hispanic populations, irrespective of sociodemographic, lifestyle, environmental, and clinical factors [97]. Among South Asians, including Indians, this higher susceptibility is explained by increased levels of insulin resistance, higher lipoprotein(a) levels, and the anatomy of coronary vessels, which are comparatively smaller [98].

**Figure 3:**
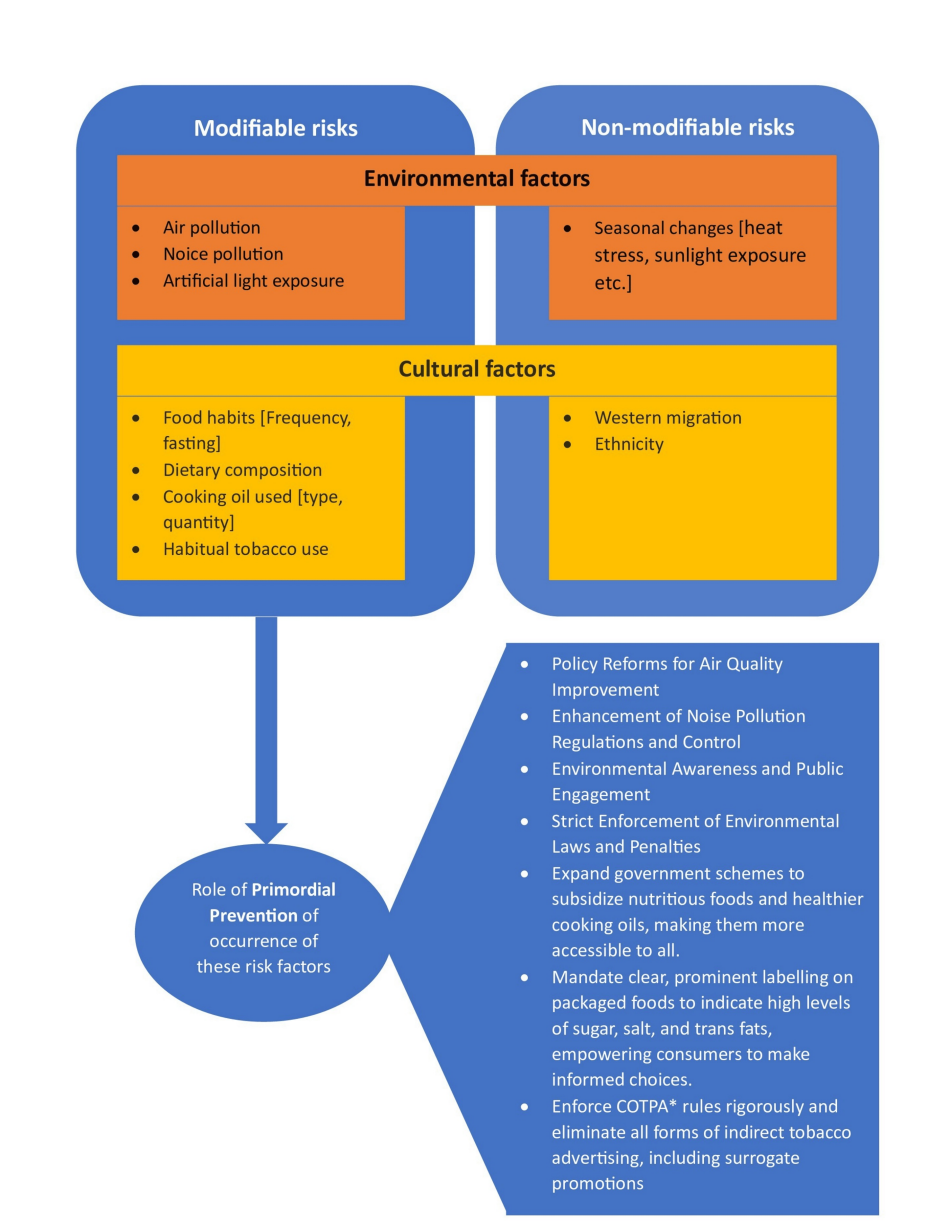
Major environmental and cultural risk factors considered in this literature review, along with their associated public health and policy implications. The image was created by the authors using Microsoft Word (Microsoft Corporation, Redmond, WA, USA).

The major limitations to be considered in this review are the difficulty in quantifying the effect of individual environmental and cultural parameters on CVD incidence, the lack of long-term longitudinal data, and limited research on cultural practices. As cultural and environmental factors vary based on different geographic regions and demographics, the focus on the Indian scenario may limit generalizability to other regions with different climatic conditions and cultural systems. Numerous factors come under the umbrella of environmental and cultural determinants, but we have considered only a main subset, due to a lack of studies. Additionally, this review was limited to studies published in English between 2010 and the present, which may have excluded relevant studies published in other languages. Despite including grey literature sources to reduce publication bias, the possibility of publication bias cannot be completely ruled out. The major environmental and cultural risk factors discussed here, along with their associated public health and policy implications, are outlined in Figure 3.

## Conclusions

Along with the conventional risk factors, various environmental and cultural factors play an important role in the causation of CVDs. Most of the environmental factors discussed above (air pollution, noise pollution, and artificial light exposure) exhibit a dose-response relationship with CVDs. The increase in the proportion of passive (second-hand) smoking has shown adverse health consequences that need to be addressed. On exploring seasonal changes, only extreme conditions (extreme heat or cold) show a significant association, rather than mild seasonal variations.

Cultural factors related to food habits were explored through meal frequency, intermittent fasting, and diet composition. As the evidence suggests, intake of three meals per day is optimal, while fewer than two or more than five meals are associated with higher CVD prevalence. Similarly, less than eight hours or more than 12-16 hours of intermittent fasting also increases the risk of CVDs. Owing to the higher oil and carbohydrate content in the traditional Indian diet, early onset of CVD-related risk factors, such as diabetes and hyperlipidemia, is identified in the population. Among the different types of cooking oils used in India, mustard oil and groundnut oil are found to be more cardioprotective, more heat-stable, and better suited for local cooking styles, followed by sunflower and coconut oil. However, as per statistics, palm oil is the most consumed oil in India due to its lower cost, and the reuse of heated oil is also common. These findings highlight the need for population-level health education and policy interventions aimed at improving awareness of appropriate types and quantities of cooking oil use. Habitual tobacco use, particularly smokeless forms prevalent in tribal populations, significantly increases CVD risk and adversely affects overall health, including perinatal outcomes such as maternal and infant mortality and low birth weight. Community-level strategies need to be planned to generate awareness and reduce the effects of tobacco use. Increased CVD risk among the immigrated Indian population is associated with lifestyle transitions and dietary patterns that are not compatible with their genetic makeup. Similarly, Asian ethnic groups exhibit a higher genetic predilection for CVDs. In conclusion, environmental and cultural risk factors play a crucial role in the development of various CVDs, which are multifactorial and linked to conventional risk factors. Addressing modifiable risk factors, such as air pollution, passive smoking, exposure to extreme seasonal conditions, and dietary habits - including meal frequency, intermittent fasting, and oil usage - can help mitigate this burden.
